# A Designed Vessel Using Dissolvable Polyvinyl Alcohol Membrane as Automatic Valve to Couple LAMP with CRISPR/Cas12a System for Visual Detection

**DOI:** 10.3390/bios13010111

**Published:** 2023-01-07

**Authors:** Tianyi Yang, Yanju Chen, Jinsong He, Jian Wu, Meixia Wang, Xiaoping Zhong

**Affiliations:** 1 College of Biosystems Engineering and Food Science, Zhejiang University, Hangzhou 310058, China; 2 College of Food Science and Technology, Yunnan Agricultural University, Kunming 650201, China; 3 ZJU-Hangzhou Global Scientific and Technological Innovation Center, No. 733, Jianshe 3rd Road, Hangzhou 311200, China; 4 Key Laboratory of Microbiol Technology and Bioinformatics of Zhejiang Province, Zhejiang Institute of Microbiology, Hangzhou 310012, China

**Keywords:** polyvinyl alcohol, dissolvable membrane, CRISPR/Cas12a, LAMP reaction, Vibrio parahaemolyticus

## Abstract

A rapid and intuitive method for detecting Vibrio parahaemolyticus (VP) was established by a designed reaction vessel which coupled CRISPR/Cas12a with loop-mediated isothermal nucleic acid amplification (LAMP). There were two spaces in the vessel-holding LAMP reaction solution and CRISPR reaction solution, respectively, which were separated with a polyvinyl alcohol (PVA) membrane. The PVA membrane could be dissolved with a water solution. The thermolabile hemolysin (TLH) gene of VP was employed as the detection target. After the target sequence of the TLH gene was amplified with LAMP, the PVA membrane would be dissolved and the CRISPR reaction solution mixed with the LAMP reaction solution. In this way, amplicons could be detected with CRISPR/Cas12a in the reaction vessel. The fluorescent signals produced by the positive samples were clearly identified by the naked eye under a UV light, while the negative samples were dark. The whole detection procedure could be finished within 35 min with a detection limit of 100 copies/µL. The designed reaction vessel is easy to produce and can effectively prevent contamination due to the opening of the reaction vessel after the LAMP reaction. Thus, it will have the potential to provide a new solution for rapid detection in the field.

## 1. Introduction

Foodborne illnesses contribute to considerable morbidity and mortality. This has become more severe due to globalization and active trade between countries [1,2]. Vibrio parahaemolyticus (VP), a Gram-negative bacterium mainly found in seawater and seafood, is a major cause of seafood-associated gastroenteritis in humans [3]. The main syndrome it causes is acute gastroenteritis. In rare cases, VP could cause wound infections and sepsis, which would be life-threatening in individuals already suffering from the disease [4]. Recently, it has been shown that VP exhibited a multiple antibiotic resistance or intermediate susceptibility test reaction to one or more antibiotic agents [5]. Therefore, it is particularly important to test food for safety, which effectively prevents foodborne illnesses at an early stage compared to treatment. Conventional methods based on cell culture for the detection of VP take about a week and are laborious [4]. In contrast, detection methods using nucleic acid amplification techniques can detect VP efficiently, such as polymerase chain reaction (PCR)-based methods [6] and loop-mediated isothermal nucleic acid amplification (LAMP)-based methods [7]. VP has some proper sequences to be amplified and detected, such as the sequences of thermolabile hemolysin (TLH). This is because TLH is not only a potential virulence factor of VP [8], but also has a conserved domain which means that it is not easily altered in inheritance.

LAMP is one of the developed isothermal amplification techniques, and it is more convenient than PCR, especially for the portable detection of DNA. The LAMP system requires 4–6 primers that can specifically identify 6–8 sites of target sequences for amplification. These primers are mixed together with buffers, dNTPs, and samples for thermostatic amplification during LAMP reactions. However, the primers of LAMP tend to produce dimers to disturb amplification. CRISPR/Cas12a can avoid the interference of primer dimers and be a visual method to detect the amplicons of LAMP.

The CRISPR-Cas system is derived from the adaptive immunity of microorganisms, and it is widely used in the field of genome editing due to its ease of use and stability [9,10]. The CRISPR/Cas12a system belongs to the V CRISPR-Cas system. The Cas12a nucleases recognize DNA target sequences with complementarity to the CRISPR RNA (crRNA) spacer, and the target sequence is positioned next to a PAM [11,12]. After crRNA binds the DNA, the indiscriminate single-stranded DNA (ssDNA) cleavage activity of Cas12a is unleashed. Therefore, the ssDNA reporters in the solution are also cut and fluoresce. This property of Cas12a can be exploited for the specific detection of DNA [13]. After the LAMP reaction is completed, the CRISPR system containing Cas12a protein, crRNA, ssDNA reporter, and buffer is mixed with the amplified products of the LAMP reaction. If the amplified products of the LAMP reaction contain the target DNA sequences, they can be detected by the CRISPR system and the ssDNA reporters’ fluoresce.

Since the enzymes in the LAMP reaction and the CRISPR reaction work at different temperatures, the reagents of the CRISPR reaction have to be added after LAMP reaction. However, to open the reaction vessel after the LAMP reaction has the risk of amplicon contamination. To avoid this problem, Zhang et al. developed an uracil-DNA-glycosylase-reverse transcription-LAMP system which could effectively remove dUTP-incorporated LAMP amplicons [14]. However, this increased the reaction time and could not prevent amplicon contamination well at large amplifications. Wu et al. reported a rotary valve-assisted microfluid chip to couple LAMP with CRISPR/Cas12a [15], but this required designing and fabricating a complex microfluid chip. There was also a dedicated reaction vessel reported where the CRISPR/Cas12a system was deposited at the vessel lid and separated from the LAMP system by a sealed membrane. The sealed membrane was poked out by a needle, which made the CRISPR/Cas12a system mix with the amplified products to conduct visual detection [16]. However, a mechanical action of poking was required after the LAMP reaction in this setup, which was not convenient and automatic.

Polyvinyl alcohol (PVA) is a copolymer prepared by the hydrolysis of polyvinyl acetate [17]. The membrane produced by PVA can be dissolved with water and the dissolution time depends on such factors as the degree of hydrolysis of the PVA, the molecular weight of the PVA, and the thickness of the membrane [18]. PVA with lower molecular weight will dissolve faster in water than PVA with higher molecular weight due to smaller intramolecular interactions [19,20]. Therefore, dissolvable PVA membranes are used in microfluidic chips [21,22], sensors [23], and 3D printing folds [24] to control the position of sample at the specified time.

**Figure 1:**
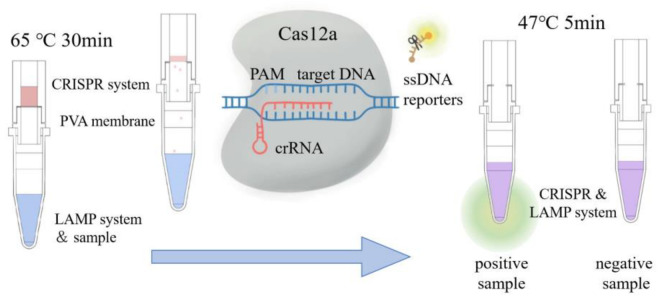
Schematic diagram of visual nucleic acid detection via LAMP-CRISPR reaction with speediness, specificity, and sensitivity.

In the present work, a dedicated vessel was designed using the dissolvable PVA membrane as an automatic valve to couple LAMP with the CRISPR/Cas12a system. It took approximately 30 min for the PVA membrane to be dissolved by the CRISPR system, during which time the LAMP reaction was performed at 65 ℃. After 30 min, the temperature of the LAMP reaction solution would be adjusted to 47 ℃. Meanwhile, the PVA membrane would be dissolved by the CRISPR solution. In this way, the CRISPR system and LAMP system were mixed, and amplicons could be detected. The fluorescent signals produced by positive samples could be clearly observed by the naked eye under UV light, while negative samples were dark. The schematic of this detection method is shown in Figure 1. This vessel effectively coupled LAMP with CRISPR/Cas12a detection and made it possible to detect VP within 35 min with specificity. It showed good ability to avoid the environmental contamination of amplicons. This vessel had the potential to be applied to other nucleic acid amplification assays. In addition, it is worth mentioning that a dedicated device was designed in order to measure the dissolution time of the PVA membranes.

## 2. Materials and Methods

### 2.1. Material

The bacterial samples were two common serotypes of VP (O3: K6 and O4: K8), which were provided by the Lin’an Center for Disease Control and Prevention (Lin’an, China). DNA was extracted from the samples using the Tiangen Bacterial Genomic DNA Extraction Kit (Tiangen Biotech Co., Ltd., Beijing, China) according to the instructions. The concentrations of extracted DNA samples were measured by Biodrop (Harvard Bioscience Inc., Holliston, MA, USA). The extraction of VP yielded a DNA concentration of 10^6^ copies/µL.

### 2.2. LAMP Reaction Process

The TLH gene (GenBank No. M36437) of VP was chosen to be the target sequences for amplification. Three sets of primers targeting the TLH gene were designed using Primer Explorer V5. Details of the LAMP primers are listed in Table 1. Three independent replicates were performed for every set, in which the mean of the experimental results was taken as the true value and the standard variance was set as the error bar.

The LAMP assay was conducted at 65 °C for 30 min. The 25 µL reaction mixture consisted of 1 × Thermol Buffer (New England Biolabs Inc., Ipswich, MA, USA), 2 mM MgSO_4_ (New England Biolabs Inc., Ipswich, MA, USA), 16 U Bst DNA polymerase (New England Biolabs Inc., Ipswich, MA, USA), 0.35 mM dNTP/dUTP Mix (Thermo Fisher Scientific Inc., Waltham, MA, USA), primer mixtures (1.6 mM FIP, 1.6 mM BIP, 0.2 mM 35S-F3, 0.2 mM B3, 0.4 mM LF, and 0.4 mM LB), 0.8 M Betaine (Sigma-Aldrich Co., LLC. St Louis, MO, USA), and 2 µL DNA solution. A real-time LAMP reaction was carried out in a QuantStudio™3 Real-Time PCR System (Thermo Fisher Scientific Inc., Waltham, MA, USA) and added with 2 µM SYTO 9 (Thermo Fisher Scientific Inc., Waltham, MA, USA).

### 2.3. CRISPR Reaction Procedure

The 20 µL CRISPR/Cas12a system contained 200 nM Cas12a protein (New England Biolabs Inc., Ipswich, MA, USA), 1 × NEB Buffer 2.1 (New England Biolabs Inc., Ipswich, MA, USA), 2.5 μM CCCCC ssDNA reporters’ probes, 100 nM crRNA (SunYa Biotechnology Co., Ltd., Hangzhou, China), 10 U RNase inhibitor (TaKara Biomedical Technology Co., Ltd., Beijing, China), and 2 µL sample solution. The sequence of crRNA is listed in Table 1. Two methods were used to observe the fluorescent signals, and one was by the real-time PCR system. The CRISPR assay was conducted at 47 °C for 30 min in the QuantStudio™3 Real-Time PCR System to record fluorescent signals. Three independent replicates were performed, in which the mean of the experimental results was taken as the true value and the standard variance was set as the error bar. Another way would be conducting the CRISPR assay at 47 °C for 5 min, and the fluorescent signals could be visualized via a homemade portable device [25].

### 2.4. Preparation of PVA Membranes

PVA was obtained from Aladdin Biotechnology Co., Ltd. (Shanghai, China). Two kinds of PVA powder (the Mv of PVA1 is 8000–10,000, the Mv of PVA2 is 85,000–1,240,000) were investigated in the study. The PVA powder was added to distilled water to obtain the solution and left to hydrate for 2 h. Afterwards, the dispersion was heated in a water bath at 90 °C for 60 min to obtain the homogeneous solution and then cooled to an ambient temperature. This method for dissolving PVA is based on the published literature [19]. Different volumes of the PVA solution (0.25 mL, 0.5 mL, 0.75 mL, and 1 mL) were poured into cell culture plates with a diameter of 1.7 cm to defoam for 10 min. After being dried in an oven at approximately 80 °C for 8 h, the plates were left at room temperature for about 20 h and demolded to obtain PVA membranes. The thickness of the PVA membrane was measured three times with a vernier caliper and then the average value was obtained.

### 2.5. A Device to Measure the Dissolution Time of PVA Membranes

**Figure 2:**
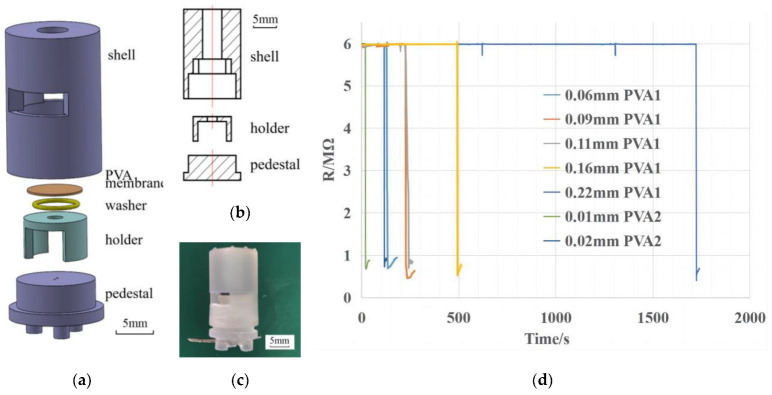
(**a**) Exploded view of the device to measure the dissolution time of PVA membranes. (**b**) Cross-sectional view of the device. (**c**) Photograph of the device. (**d**) Resistance curve of different kinds of membranes with time.

In order to easily and accurately measure the dissolution time of the PVA membranes, a device was designed as shown in Figure 2. It was made from resin and prepared by 3D printing. The solution to dissolve the PVA membrane was stored above the membrane which was cut into a regular-sized disc. Two electrodes were inserted in the pedestal, which were connected to a multimeter and a computer to measure the resistance between electrodes in real time. When the PVA membrane was not dissolved, there was an open circuit between two electrodes. When the PVA membrane was dissolved, the solution would drop. In this case, two electrodes would be immersed in the solution and the resistance obtained dropped sharply. The time at which the resistance changed markedly could be obtained. Three independent replicates were performed, in which the mean of the time was taken as the true value and the standard variance was set as the error bar. Therefore, we could accurately measure the time required for the PVA membrane to be dissolved.

### 2.6. A Dedicated Vessel for the LAMP-CRISPR Reaction

**Figure 3:**
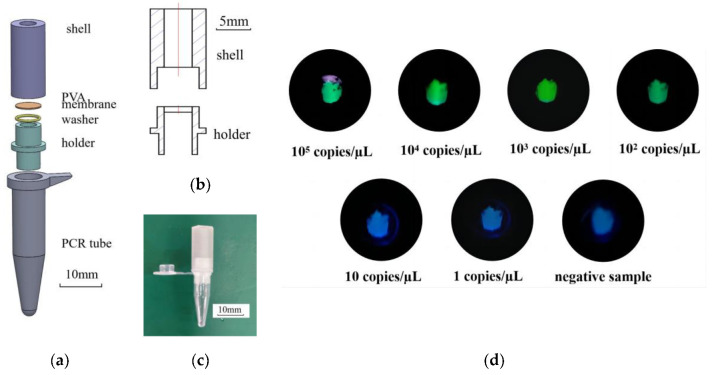
(**a**) Exploded view of the dedicated vessel for LAMP-CRISPR reaction. (**b**) Cutaway view of the dedicated vessel. (**c**) Photograph of the dedicated vessel. (**d**) The DNA solution of gradient concentration and solution for reaction were placed in the vessel. Visualized fluorescent signals can be obtained after 30 min of LAMP reaction and 5 min of CRISPR reaction.

If the temperature exceeded 60 °C, the activity of the Cas12a protease would decrease significantly. Thus, the CRISPR system should be added only after the LAMP reaction was finished. However, additional intervention is often required between the two reactions, such as opening the lid and mixing solutions. That is not only inconvenient, but also prone to amplicons’ contamination. Therefore, a resin vessel was prepared by 3D printing to solve this problem, which is shown in Figure 3a. The LAMP solution and the CRISPR solution were separated by a PVA membrane that could be dissolved at certain intervals. At the end of the LAMP reaction, the CRISPR system above dissolved the PVA membrane and dropped into the PCR tube, triggering the CRISPR reaction.

## 3. Results and Discussion

### 3.1. LAMP Reaction for Detection of VP

**Figure 4:**
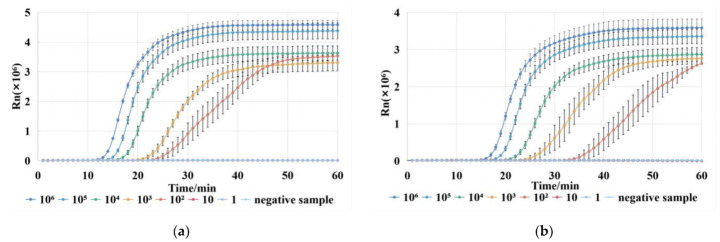

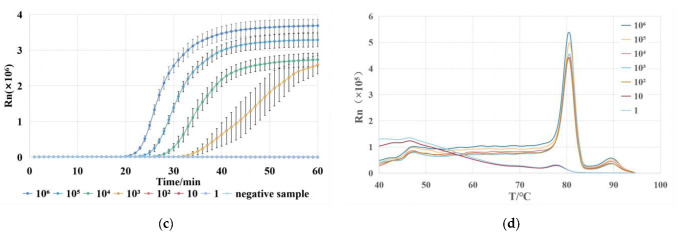
(**a**) Fluorescent signals of the first set of LAMP reactions for gradient dilution of VP DNA extracts of 10^6^ copies/μL. (**b**) Fluorescent signals of the second set of LAMP reactions. (**c**) Fluorescent signals of the third set of LAMP reactions. (**d**) Melting curves of the amplified products of the first set of LAMP reactions.

In this work, three different sets of LAMP primers were designed. The results of the LAMP reaction are shown in Figure 4. The minimum detectable DNA concentration was 100 copies/µL for the first and second sets, and 1000 copies/µL for the third set. The fluorescent signals obtained from the first set of primers were higher, and the fluorescent signals of all target concentrations could start to increase within 30 min. Therefore, the first set of primers was chosen for the LAMP reaction. In addition, we analyzed the amplified products of the first set of the primers by the melt curves shown in Figure 4d. It can be found that there is only a clear peak at 82 °C, which indicates the melting temperature of the amplicons. Thus, the first set of primers in the LAMP reaction has good specificity.

### 3.2. CRISPR/Cas12a Reaction for Detection of VP

**Figure 5:**
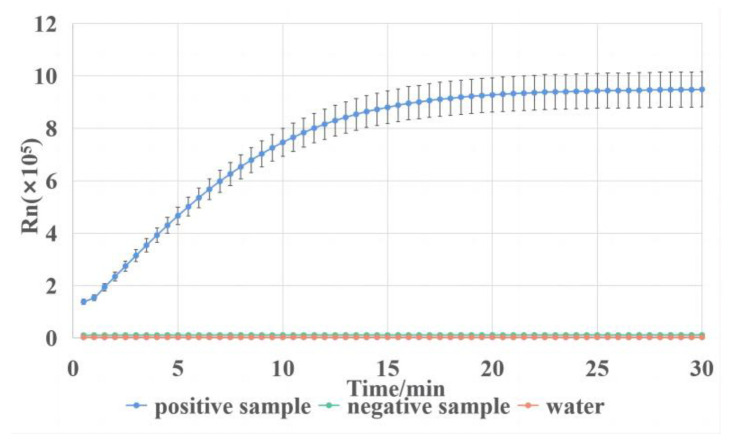
Fluorescent signals of CRISPR reaction on LAMP reaction products using the first set of primers.

To be able to visualize the results of the LAMP reaction, we chose to detect the amplicons by the CRISPR/Cas12a system. This method prevents the interference of a dimer generated by the LAMP primers and improves the accuracy and specificity of the detection. The fluorescent signals obtained from the CRISPR reactions are shown in Figure 5. The samples for the CRISPR reaction were pure water, the positive sample, and the negative sample. Furthermore, the positive sample was the amplified products of the sample with a DNA content of 100 copies/µL, while the negative sample was the amplified products of pure water. The result shows that only the positive LAMP reaction products were recognized by the CRISPR reaction, and the fluorescent signal reached the plateau within 10 min. Therefore, the CRISPR/cas12a system could successfully detect the LAMP amplicons of VP. In summary, the LAMP-CRISPR system can detect VP with high efficiency and specificity.

### 3.3. Investigation of Solubility Characteristics of PVA Membranes

In order to mix the CRISPR solution with the LAMP solution after approximately 30 min, the PVA membrane which could be dissolved by the CRISPR solution in 30 min was required. Membranes made from two different kinds of PVA were investigated. PVA membranes were obtained by injecting the homogeneous PVA aqueous solution into cell culture plates with a diameter of 1.7 cm. A too high mass fraction of the PVA solution (PVA1 higher than 16% and PVA2 higher than 8%) would result in membranes with air bubbles, while a too low mass fraction would result in thin and fragile membranes. It was found that 8% PVA1 solution and 4% PVA2 solution were suitable for PVA membrane preparation. Too little PVA solution would not cover the bottom of the cell culture plates. However, if too much PVA solution was added, the surface layer of the solution would first form a membrane during the drying process. This not only resulted in membranes with uneven thickness and surface, but also increased the time required for the drying process. The upper limit of the thickness of PVA1 membranes was 0.16 mm, while that of PVA2 membranes was 0.02 mm. Therefore, PVA1 was prepared into an 8% solution and dried to obtain 0.06 mm, 0.09 mm, 0.11 mm, and 0.16 mm thin membranes (prepared by 0.25 mL, 0.5 mL, 0.75 mL, and 1 mL PVA1 solution, respectively). PVA2 was prepared into a 4% solution and dried to obtain 0.01 mm and 0.02 mm thin membranes (prepared by 0.25 mL and 0.5 mL PVA2 solutions, respectively).

The PVA membranes were cut into discs with a diameter of 6 mm and placed between the holder and the shell in the device shown in Figure 2a. A tubular space existed above the PVA membrane to hold the CRISPR system for dissolving the PVA membrane. The friction between the pedestal and the shell was used to be able to compress the PVA membrane and the washer in order to prevent water from leaking out. Two small vertical holes were left in the pedestal for inserting electrodes connected to the multimeter and the computer. After installation in the order of Figure 2a, the solution was added while starting the timing. At the beginning, there was an open circuit between the electrodes. When the PVA membrane above was dissolved and broken, the solution flowed between the two electrodes, and the resistance between electrodes became dramatically smaller which could be detected by the multimeter. In order to increase the accuracy of the measurement, a 6 MΩ resistor was connected in parallel between two electrodes avoiding measuring the open resistance. The point at which the resistance dropped rapidly was the point at which the PVA membrane was dissolved by the solution, as shown in Figure 2d.

**Figure 6:**
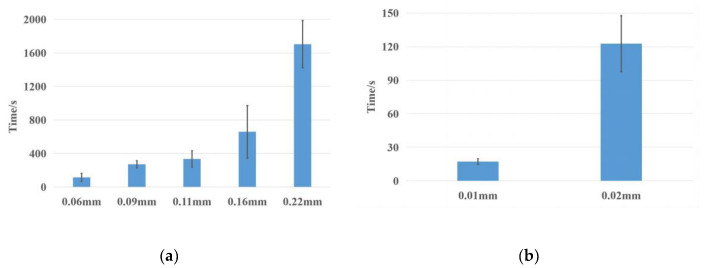
(**a**) Dissolution time of PVA1 membranes with different thickness, where the membrane marked 0.22 mm is a superposition of two 0.11 mm membranes. (**b**) Dissolution time of PVA2 membranes with different thickness.

With this device, six kinds of PVA membranes were investigated and the dissolution time was obtained as shown in Figure 6. It could be found that the thicker the PVA membrane, the longer it took to dissolve. The dissolution time of PVA1 membranes could reach about 10 min at most, and that of PVA2 membranes could reach 2 min at most. Therefore, an 8% PVA1 aqueous solution was more suitable for making membranes to control the LAMP reaction time. Since the dissolution time of the 0.16 mm PVA1 membrane remained less than 30 min, two membranes of the same thickness were chosen to be stacked to increase the dissolution time. Due to the smooth surface of the PVA membranes and water-soluble property, the two layers of the PVA membrane could be better laminated to a bubble-free state by pressing. The experimental results showed that two 0.11 mm PVA1 membranes could be stacked to obtain a double-layer membrane with a dissolution time of about 30 min.

### 3.4. LAMP-CRISPR Application for VP Testing

In order to couple LAMP with CRISPR detection and avoid environmental contamination by amplicons of the LAMP reaction, a vessel for the LAMP-CRISPR reaction was designed. The vessel consisted of four main parts, which were the holder, shell, washer, and a PVA membrane, as shown in Figure 3a. The holder was used to support the PVA membrane. The hole in the middle of the holder allowed the CRISPR reaction system above to drip smoothly into the PCR tube below after the PVA membrane was dissolved. The holder was directly connected to the PCR tube which served as a vessel for the LAMP reaction. The washer between the holder and the PVA membrane increased the sealability of the vessel. Furthermore, it prevented the CRISPR reaction solution above from leaking out, and also avoided the environmental contamination of amplicons. The shell and the PVA membrane together formed a tubular space for storing the CRISPR solution. The shell was sealed with tape to avoid environmental contamination. This vessel not only effectively coupled LAMP and CRISPR systems but also effectively isolated the reaction from the environment.

In the process of using the dedicated vessel for the LAMP-CRISPR reaction, the LAMP reaction solution with the DNA extract was added in the PCR tube below. The PVA membrane was cut into a disc of 6 mm in diameter and placed between the holder and the shell with a washer. After pressing firmly against the holder and PCR tube, the 40 µL CRISPR reaction solution was placed on the PVA membrane. Then, this vessel was sealed with tape at the top and placed on a metal heat block at 65 °C. After 30 min of reaction, the temperature was adjusted to 47 °C automatically. At the same time, the CRISPR solution above dissolved the PVA membrane and dropped into the PCR tube below, triggering the CRISPR reaction. After 5 min of the CRISPR reaction, under UV light, clear fluorescence appeared in the PCR tube with positive samples, while the PCR tube with negative samples was dark. The DNA solution of gradient concentration was tested in the vessel. The detection limit of VP was 100 copies/µL, as shown in Figure 3d.

In the process of using this vessel for food contamination detection, DNA needed to be extracted. Since VP is usually found in seafood, shrimp is a good choice as a sample. In our previous work [26], a 30 mg sample of shrimp was ground and added sodium hydroxide solution to produce a DNA extract. The process of extraction took only two minutes. Then, the extracts needed to be detected to show if the DNA of VP was present. Compared with existing methods for VP detection [27,28], the whole detection procedure could be finished within 35 min with a detection limit of 100 copies/µL. In this dedicated vessel, the PVA membrane effectively coupled the LAMP reaction with the CRISPR detection, allowing the CRISPR reaction to occur automatically 30 min after the LAMP reaction and avoid the environmental contamination of amplicons. The difference between the positive (green fluorescence) and negative (dark) samples can be clearly observed by the naked eye under the UV light. This vessel provides a possible solution for rapid nucleic acid detection in the field.

## 4. Conclusions

In this study, a vessel was designed for the LAMP and CRISPR reaction to detect VP with rapidity and visuality. There were two spaces in the vessel holding a LAMP reaction solution and CRISPR reaction solution, respectively, which were separated with a PVA membrane. After the target sequence of VP was amplified with LAMP, the dissolved PVA membrane triggered the CRISPR reaction. In this way, amplicons could be detected by the CRISPR/Cas12a system in the reaction vessel. Positive samples emitted green fluorescence under UV light, which could be recognized by the naked eye. The whole detection procedure finished within 35 min, and the detection limit reached 100 copies/µL. It was necessary to use the PVA solution of the appropriate mass fraction (8% PVA1 solution and 4% PVA2 solution) to produce uniform and stable membranes. The dissolution time of PVA membranes could be tested with a dedicated device. The dissolution time of the double-layer membrane obtained by two 0.11 mm PVA1 membranes was 30 min, which was well suited to the dedicated vessel.

## Figures and Tables

**Table 1:** Sequences of primers and crRNA.

Category	Primer	Primer Sequences (5′to3′)
The first set of primers used in LAMP assay	TLH1-F3	ATCGCACCAGCTACTCGA
	TLH1-B3	CGGCGAAGAACGTAATGTCT
	TLH1-FIP	CCACCAGTAGCCGTCAATGGTGAAGATGATCCAGCGACCGAT
	TLH1-BIP	ACACCAACACGTCGCAAAACGGCGTTCTCGTTCGCCAAAT
	TLH1-LF	TCGTTTTTTGCCCATTCCCA
	TLH1-LB	TTATCCGTCAGCGTTGTGAAGC
The second set of primers used in LAMP assay	TLH2-F3	TCGTACTTAACCTACGCAA
	TLH2-B3	TGAGTACTTAAACTGAGGCG
	TLH2-FIP	CGGTTGTAGTTCATGAAGTCATTCAACTGGCGAAGAACTACAAAC
	TLH2-BIP	GTTCCAGAAGTGAAAGCGGATTATCAGTGTCATCAACATGAAGTT
	TLH2-LF	GCGTAAACAAGGTGTTTGCTG
	TLH2-LB	AGCACTGATTCGTTTGACGGA
The third set of primers used in LAMP assay	TLH3-F3	CCGAAGAGCACGGTTTCG
	TLH3-B3	CGGTACTCGGCTAAGTTGTT
	TLH3-FIP	CGCAATGCGTGGGTGTACATGTTGAACGCGAGCGATCCTT
	TLH3-BIP	GTGTGCAGCGTCTGGTGCTGCTGCAACATAGCGGTGAGT
	TLH3-LF	CGATGAGCGGTTGATGTCCA
	TLH3-LB	GAAGTTTGTGTTCTGGAATGTCAC
crRNA used in CRISPR/Cas12a	TLH1-crRNA	UAAUUUCUACUGUUGUAGAUAAUGAAACGGAGCUCCACCAG

## Data Availability

Not applicable.
